# Correlations Between MRI Biomarkers PDFF and cT1 With Histopathological Features of Non-Alcoholic Steatohepatitis

**DOI:** 10.3389/fendo.2020.575843

**Published:** 2021-01-27

**Authors:** Andrea Dennis, Matt D. Kelly, Carolina Fernandes, Sofia Mouchti, Jonathan A. Fallowfield, Gideon Hirschfield, Michael Pavlides, Stephen Harrison, Manu V. Chakravarthy, Rajarshi Banerjee, Arun Sanyal

**Affiliations:** ^1^ Perspectum, Oxford, United Kingdom; ^2^ Centre for Inflammation Research, University of Edinburgh, Edinburgh, United Kingdom; ^3^ Toronto Centre for Liver Disease, University Health Network, Toronto, ON, Canada; ^4^ Radcliffe Department of Medicine, University of Oxford, Oxford, United Kingdom; ^5^ Translational Gastroenterology Unit, University of Oxford, Oxford, United Kingdom; ^6^ Oxford NIHR Biomedical Research Centre, University of Oxford, Oxford, United Kingdom; ^7^ Pinnacle Clinical Research, San Antonio, TX, United States; ^8^ Axcella Health, Cambridge, MA, United States; ^9^ Division of Gastroenterology, Hepatology and Nutrition, Department of Internal Medicine, Virginia Commonwealth University, Richmond, VA, United States

**Keywords:** imaging, NASH, NAFLD, non-invasive biomarkers, MRI

## Abstract

**Introduction:**

Late stage clinical trials in non-alcoholic steatohepatitis (NASH) are currently required by the FDA to use liver biopsy as a primary endpoint. The well-reported limitations with biopsy, such as associated risks and sampling error, coupled with patient preference, are driving investigation into non-invasive alternatives. MRI-derived biomarkers proton density fat fraction (PDFF) and iron-corrected T1 mapping (cT1) are gaining traction as emerging alternatives to biopsy for NASH. Our aim was to explore the correlations between cT1 and PDFF (from Liver*MultiScan*®), with the histological components on the NAFLD-NASH spectrum in a large cohort of cross-sectional data, in order to calibrate the measurement to histology, and to infer what might constitute a clinically meaningful change when related to the FDA’s criteria.

**Materials and Methods:**

In a retrospective analysis of data combined from three previously published observational NASH studies, in which adult participants who underwent liver biopsy on suspicion of NAFLD or NASH and had an MRI scan measuring cT1 and PDFF (Liver*MultiScan*®, Perspectum Ltd, UK), associations between imaging biomarkers and histology were tested using Spearman’s rank correlation coefficient (r_s_), and further exploration of the relationships between the imaging variables and histology were performed using linear regression.

**Results:**

N = 264 patients with mean age of 54 (SD:9.9), 39% female, and 69% with BMI ≥ 30kg.m^−2^ were included in the analysis. cT1 and PDFF both correlated with all features of the NAFLD activity score (NAS). cT1 was also positively correlated with Kleiner-Brunt fibrosis. Partial correlations, adjusting for steatosis, revealed cT1 correlated with inflammation and fibrosis, whereas PDFF did not, and both were still associated with the NAS, but correlation was weaker with PDFF than cT1. An estimated difference of 88 ms in cT1, or 21% relative difference in PDFF was related to a two-point difference in overall NAS.

**Conclusion:**

The correlations between cT1 and PDFF with the histopathological hallmarks of NASH demonstrate the potential utility of both cT1 and PDFF as non-invasive biomarkers to detect a pharmacodynamic change in NASH, with cT1 showing superiority for detecting changes in inflammation and fibrosis, rather than liver fat alone.

## Introduction

Non-alcoholic fatty liver disease (NAFLD) and its progressive form non-alcoholic steatohepatitis (NASH) have complex histological signatures reflecting coexisting fat deposition, inflammation, hepatocellular injury (ballooning) and fibrosis, that are each subject to time-dependent and reversible changes. NASH results when fat accumulation in the liver triggers inflammatory signals and reactive oxygen species that can amplify liver injury and stimulate fibrosis (1). NAFLD is the most common cause of chronic liver disease in the world, affecting approximately 25% of the global population (2), with a quarter of those having NASH, or approximately 6% of the general population worldwide (3). NASH is now the second most common cause for liver transplantation in the US overall (4) and is the leading cause in females (5). This clinical burden has driven a rapid increase in the number of clinical trials evaluating pharmacotherapies. Liver biopsy is the current gold standard measurement for both clinical diagnosis and as endpoints in clinical trials, a method that is expensive, invasive, and suffers from high discordance rate among pathologists (6), likely related to the uneven distribution of the disease (7). This has driven the need to identify alternative, non-invasive, endpoints which the FDA has strongly encouraged (8). Vendor-neutral and scalable MRI-derived measurements of proton density fat fraction (PDFF) and iron corrected T1 mapping (cT1) are emerging as promising quantitative imaging biomarkers (QIBs) for NASH.

PDFF has an excellent correlation with histologically graded steatosis across the clinical range seen in NASH (9–11) and high diagnostic accuracy in stratifying all grades of liver steatosis (12–14), although it decreases with advanced fibrosis (9). PDFF is a more robust method of measuring liver fat than histology (7, 15), has been shown to be a repeatable and reproducible metric (16–18) that is sensitive to small changes (15), and as such it is considered to be the most superior non-invasive test for liver fat (19). cT1 mapping is an indicator of regional tissue water content. Each of the key histopathological features of NASH is known to have an influence on the cT1 signal when measured using the T1 “shMOLLI” sequence (20–22); as such, is it has been reported to correlate with ballooning (23, 24), fibrosis (22, 23, 25, 26), and NAS (24), and has also been shown to predict clinical outcomes (27). Liver cT1 has also been shown to be significantly elevated in patients with clinically significant portal hypertension with low liver fat (28), and has also been reported to be repeatable and reproducible across MRI manufacturers and field strengths (17).

A number of phase II trials are already employing the non-invasive QIBs PDFF and cT1 as diagnostic screening biomarkers and as secondary or exploratory endpoints (29–31). In order for QIBs to be adopted as primary endpoints in pivotal trials, they must demonstrate agreement with liver biopsy and ability to measure a clinically meaningful response. Meaningful responses are being classified as those demonstrating either (i) a resolution of steatohepatitis as defined by a ballooning score of 0 and an inflammation score of 0–1 and no worsening of liver fibrosis, (ii) improvement in liver fibrosis greater than or equal to one stage and no worsening of steatohepatitis, or (iii) both resolution of steatohepatitis and improvement in fibrosis (8), often expressed as a two-point change in the NAFLD activity score (NAS) with no worsening in fibrosis. Analysis of a trial of ezetimibe in NASH showed that PDFF could distinguish histological responders from non-responders (32), which was later characterized as a relative reduction of ~30% liver fat corresponding to a two-point change in NAS (33, 34). Equally, data on the ability of cT1 to measure changes in fibroinflammatory disease have recently been published (30, 31). In a trial exploring the efficacy of an FGF-19 (fibroblast growth factor) analog in patients with NASH (NCT02443116), patients showed significant drops in both PDFF and cT1, with greater reductions in cT1 (as well as in circulating biomarkers of fibrosis, ELF, and Pro-C3) (30). Specifically, it was observed that a reduction in cT1 of 78 and 82 ms in the 1- and 3-mg treatment groups, respectively, accompanied the regulatory accepted histological response. Furthermore, analysis comparing histological responders from non-responders showed greater reductions in cT1 than in PDFF following 12 weeks of therapy (30).

In order to inform what drives change in cT1 and PDFF, and also to estimate what might constitute a meaningful change in both biomarkers when compared to biopsy, we set out to explore the relationships between both QIBs and the histological features of NASH in a cross-sectional analysis of a large cohort of NAFLD patients with paired biopsies and MRI scans.

## Experimental Procedures

### Study Design and Setting

**Figure 1:**
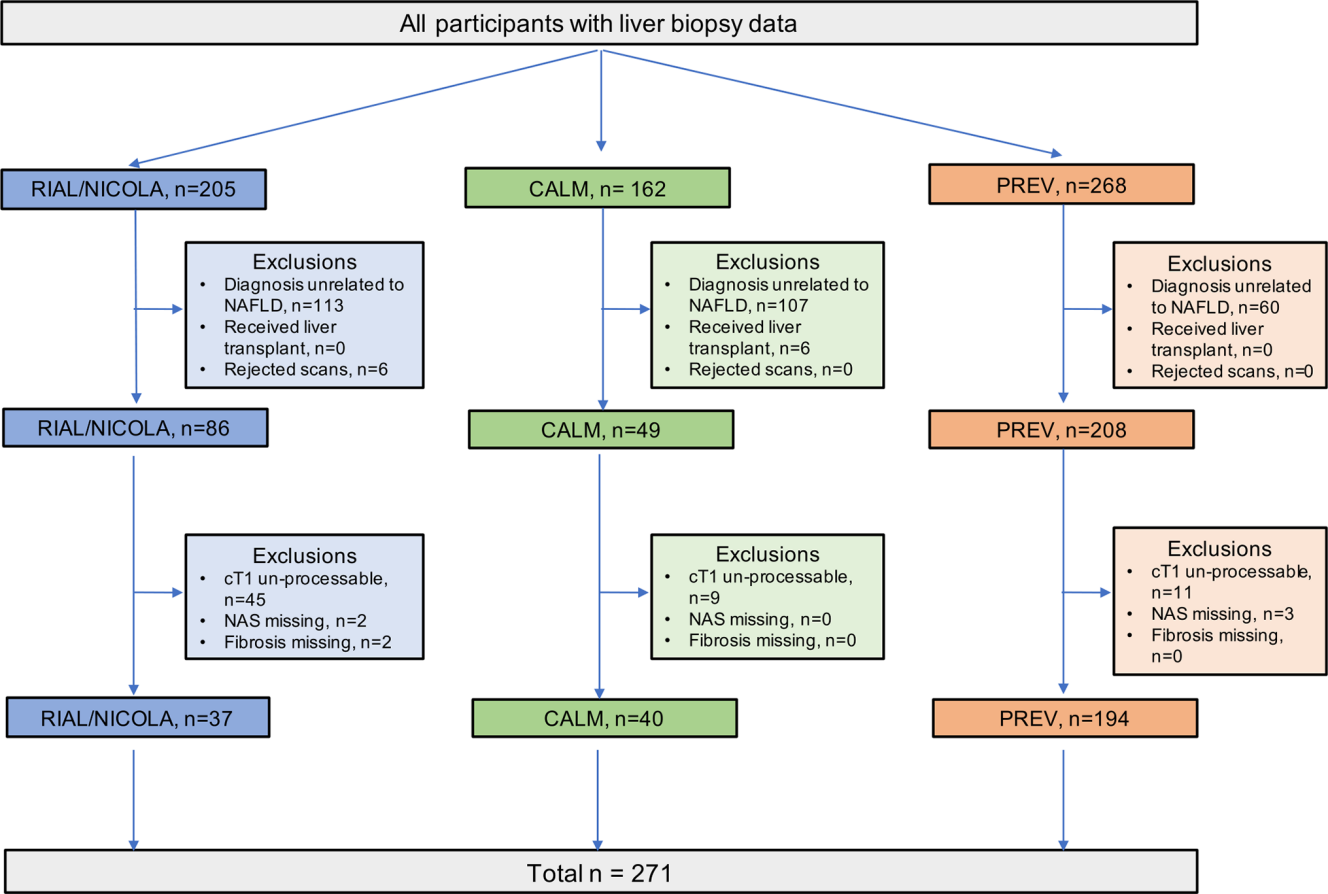
Flow diagram of patient inclusion from the three trials RIAL/NICOLA, CALM, and PREVALENCE.

This was a retrospective analysis of a subset of data combined from three prospective, cross-sectional studies on the utility of MR methods to evaluate liver disease. The RIAL (NCT01543646)/NICOLA study enrolled adult patients scheduled for a standard-of-care liver biopsy to investigate known or suspected liver disease from two large tertiary UK liver centres (Oxford and Reading) between March 2011 to May 2015. Similarly, the CALM study (ISCRTN39463479) invited adult patients scheduled for a standard-of-care liver biopsy to investigate known or suspected liver disease from two large tertiary UK liver centres (Queen Elizabeth Hospital Birmingham and Royal Infirmary of Edinburgh) between February 2014 and September 2015 (26). Patient exclusion criteria for both RIAL/NICOLA and CALM studies were the same: inability or unwillingness to give fully informed consent, any contraindication to MRI, and liver biopsy targeted at a focal liver lesion. Full details have been published elsewhere (25, 26). For the purpose of this analysis, only those patients from both studies with a histological diagnosis of NAFLD were included (**Figure 1**). The Prevalence study (NCT03142867) invited adults who were being screened for colon cancer to participate in a trial to investigate the prevalence of NAFLD at Brooke Army Medical Center in San Antonio, Texas, between August 2015 and December 2017. Participants had no prior history of liver disease or alcohol abuse. Liver*MultiScan* ^®^, FibroScan^®^ liver stiffness measurement (LSM) with controlled attenuation parameter (CAP) and MR Elastography (MRE) were acquired as part of the screening protocol. Participants were invited to undergo core liver biopsy if evidence of steatosis (PDFF ≥ 5%) or fibro-inflammation (from one of LSM ≥ 7.0 kPa, evidence of fibrosis on MRE, elevated cT1 ≥ 780ms). Full details have been described elsewhere (35). All studies were conducted in accordance with the ethical principles of the Declaration of Helsinki 2013, were approved by local (all) and or national (RIAL: 11/H0504/2 and CALM 14/WM/0010 ethics review services), and all participants gave written informed consent.

### Histological Analysis of Liver Biopsy Samples

All biopsies were reported by at least two liver histopathologists, and adequacy assessed using the definition of the Royal College of Pathologists, UK. Histology was graded according to the NASH-CRN for Kleiner-Brunt fibrosis, hepatocellular ballooning, lobular inflammation, steatosis, and the composite NAS. All pathologists were blinded to patient characteristics and non-invasive assessment data. Discordance was adjudicated by a third blinded observer. Biopsy scores used for the analysis were those collected as part of the three independent studies and were not re-read centrally.

### Magnetic Resonance Protocol and Analysis

The Liver*MultiScan* ^®^ MRI scanning protocol was installed, calibrated, and phantom tested on all the MRI systems in these trials in a standard way (17). Patients underwent their MRI having fasted for at least 4 h. The average scan time for this protocol was 10 min. The MRI protocol included a multi-echo spoiled-gradient-echo chemical shift encoded acquisition to calculate T2* and PDFF maps in most cases, although some PDFF values were generated using *in vivo* proton magnetic resonance spectroscopy (MRS), a specialized magnetic resonance technique that measures fat by quantifying the overall volume fraction of lipids in the liver parenchyma. A ShMOLLI sequence was used to derive T1-relaxation. Iron was calculated from the T2* relaxation. To generate cT1 maps, the acquired MOLLI image data were fit using a Bloch equation simulation approach. The resultant cT1 maps were generated according to the latest Liver*MultiScan* ^®^ post-processing algorithms that reflect a measurement that would be expected if the patient had been scanned with a heart rate of 60 bpm, with normal liver iron and on a Siemens 3T scanner. This general approach has been found to yield accurate fitting to MOLLI data in previous work [as described by Mozes et al. (21)] and has been shown to improve standardization across vendors and field strengths by standardizing the contribution from fat. As a result a higher proportion of MRI scans from the older trials did not pass the required data quality checks for this processing and thus were excluded from further analysis (**Figure 1**).

**Figure 2:**
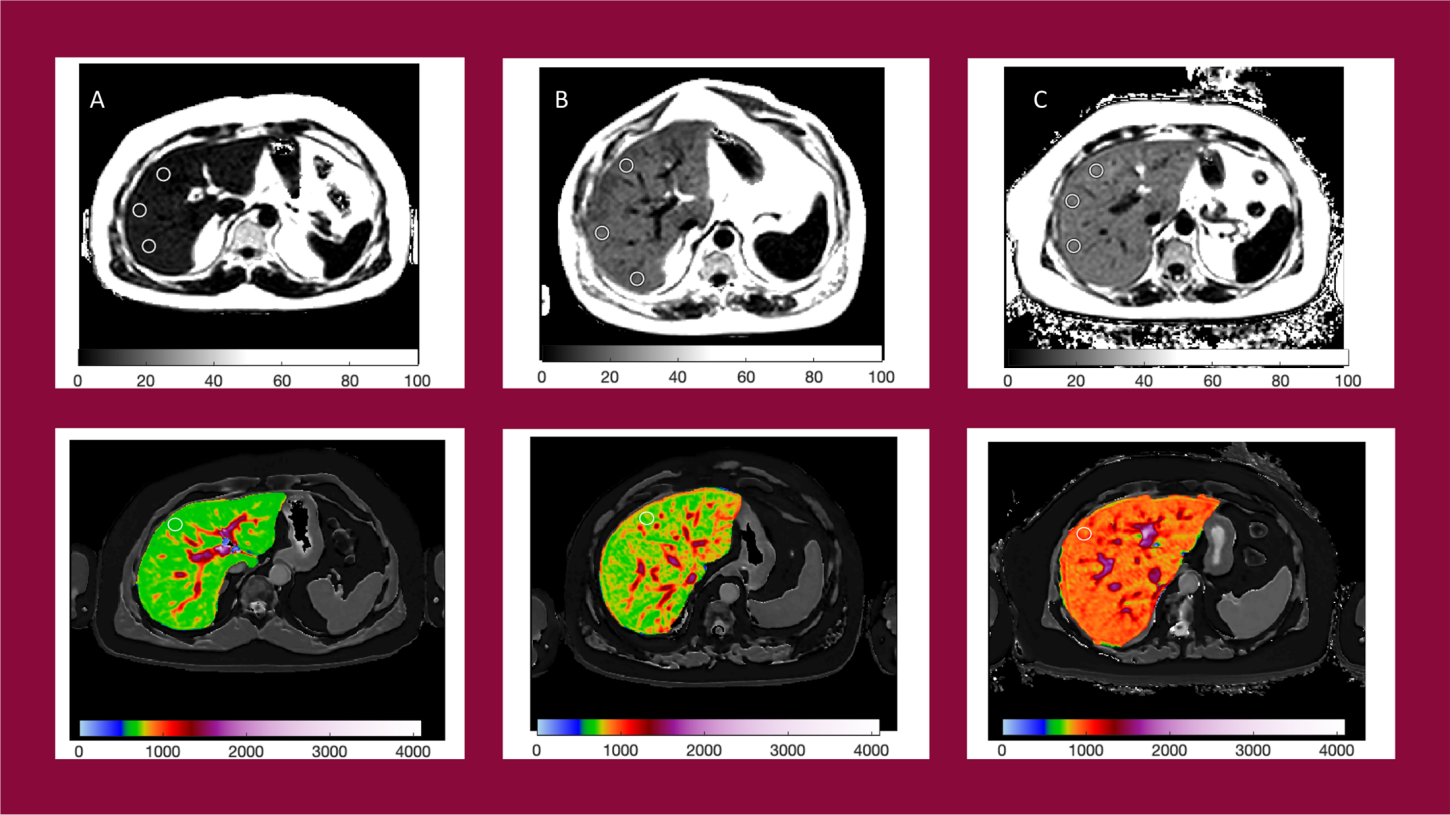
Example PDFF and cT1 parametric maps for patients with NAS = 1 **(A)** cT1 = 684ms, PDFF = 6.5%; NAS = 3 **(B)** cT1 = 833ms, PDFF = 16.9%; and NAS = 5 **(C)** cT1 = 916ms, PDFF = 18.5%.

Four single transverse slices were captured through the liver centred on the porta hepatis. Anonymized MR data were analyzed off-site using Liver*MultiScan* ^®^ software by image analysts trained in abdominal anatomy and artifact detection, who were blinded to the clinical data and risk grouping. For T2* (measured in milliseconds, ms) and PDFF (measured in %), three 15-mm diameter circular regions of interest (ROIs) were selected on the transverse maps to cover a representative sample of the liver parenchyma. For cT1 (ms), ROIs were placed on the central slice within the typical percutaneous biopsy region. Median values from all pixels within the ROIs were calculated and used as the representative score. Example images are presented in **Figure 2**.

### Statistical Analysis

Descriptive statistics were used to summarize baseline participant characteristics. Mean and standard deviation (SD) were used to describe normally distributed continuous variables, median with interquartile range for non-normally distributed, and frequency and percentage for categorical variables. The distribution of the QIB data and the categorical histological data was investigated using box plots.

Associations between both QIBs and histology were tested using the Spearman’s rank correlation coefficient (_rs_), both with and without adjusting for steatosis as a covariate. A p-value less than 0.05 was considered statistically significant.

Further exploration of the relationships between the dependent QIB variables and histological scores for NAS, fibrosis and ballooning as the explanatory variables were performed with linear regression analysis, following confirmation of assumptions of normality in the residuals (and log transforming parameters that did not meet these assumptions). Extraction of the difference in imaging biomarkers associated with the histological endpoints (i) two-point difference in NAS and (ii) 1 stage difference in ballooning (in order to predict level required per patient to return to stage 0 depending on ballooning at baseline) were performed by applying the equation for each linear model.

Case-wise deletion was employed to include only complete cases for NAS, Kleiner-Brunt Fibrosis score and cT1 and PDFF data, and to exclude NAS groups with less than five data points. All statistical analysis was performed using R software version 3.6.0.

## Results

Following case-wise deletion, a total of 264 biopsy and MRI paired datasets were included in the analysis (N = 187 from prevalence study, N = 40 from CALM, and N = 37 from RIAL/NICOLA). The mean (SD) age was 54 years (9.7) and patients had a median Body Mass Index (BMI) of 32.6 kg.m^−2^ (IQR 29.7–36.3). The majority of the patients were male (n = 161; 61%). The participant characteristics of the whole cohort (demographics, histology and MR data) are presented in **Table 1**.

**Table 1:** Baseline patient characteristics.

N = 264	Statistic
Age (years; mean [SD])	54.1 [9.6]
Sex (F, %)	103 (39%)
BMI (kg.m^−2^; median; IQR)	32.6 (29.7–36.3)
Fibrosis (n,%) F0	83 (31)
F1	96 (36)
F2	42 (16)
F3	31 (12)
F4	12 (5)
Ballooning (n,%) B0	92 (35)
B1	128 (48)
B2	44 (17)
Lobular Inflammation (n,%) I0	72 (27)
I1	158 (60)
I2	32 (12)
I3	2 (1)
Steatosis (n,%) S0	0 (0)
S1	119 (45)
S2	80 (30)
S3	65 (25)
NAS (n,%) 1	38 (14)
2	50 (19)
3	42 (16)
4	43 (16)
5	69 (26)
6	17 (6)
7	5 (2)
NASH classification* (n,%) NAFL	175 (66%)
NASH	89 (34%)
cT1 (mean ms; [SD]) NAFL	836.3 [125]
NASH	859 [108]
PDFF (mean %; [SD]) NALF	9.7 [6.3]
NASH	14.3 [5.8]

*NASH classification using either the FLIP [fatty liver inhibition of progression, (36)] algorithm or the CRN criteria depending on availability.

### Correlations Between Variables

**Figure 3:**
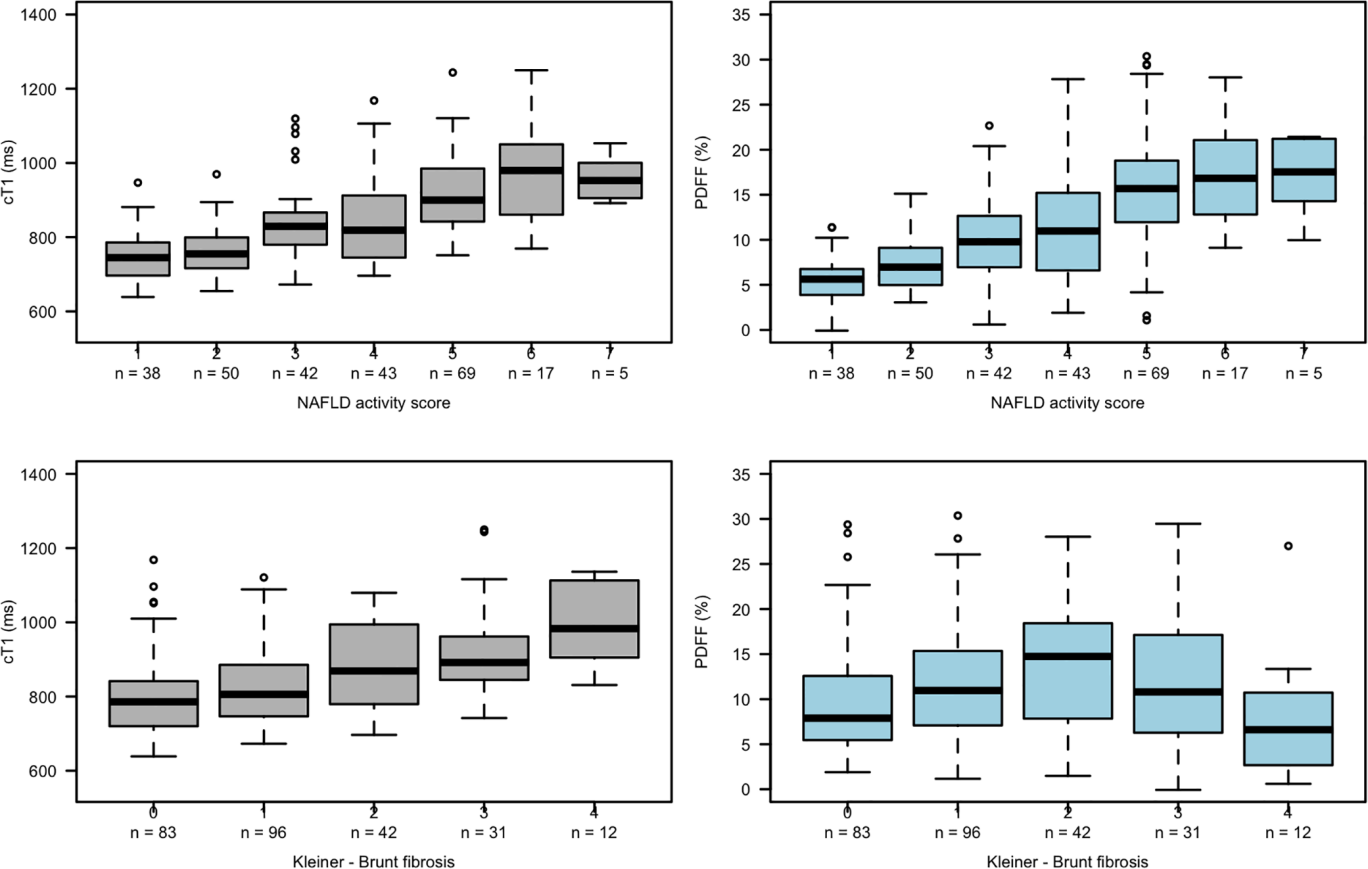
Box plots showing relationships between cT1 and PDFF with NAS (top row) and fibrosis (bottom row).

Box plots (**Figure 3**) showing the relationships between NAS and fibrosis with both cT1 and PDFF indicate a positive linear relationship between cT1 and both NAS and fibrosis, and between PDFF and NAS. However, the relationship between PDFF and fibrosis showed a more parabolic distribution, reflecting the common observation that patients with severe fibrosis often have lower liver fat, and thus preventing meaningful interpretation of linear associations.

The correlations between all imaging and histological variables are shown in **Table 2**. All histological parameters were correlated with each other.

**Table 2:** Spearman’s correlation coefficients for all variables.

N = 264		Steatosis	Ballooning	Inflammation	Fibrosis	NAS
Steatosis			rs = 0.38, P <.001	rs = 0.31, P <.001	rs = 0.19, P <.001	rs = 0.78, P <.001
Ballooning				rs = 0.39, P <.001	rs = 0.44, P <.001	rs = 0.77, P <.001
Inflammation					rs = 0.43, P <.001	rs = 0.71, P <.001
Fibrosis						rs = 0.45, P <.001
cT1	Full	rs = 0.54, P <.001	rs = 0.47, P <.001	rs = 0.31, P <.001	rs = 0.43, P <.001	rs = 0.59, P <.001
	Partial		rs = 0.36, P <.001	rs = 0.17, P <.05	rs = 0.33, P <.001	rs = 0.36, P <.001
PDFF	Full	rs = 0.68, P <.001	rs = 0.38, P <.001	rs = 0.28, P <.001	rs = 0.08, P <.05	rs = 0.61, P <.001
	Partial		rs = 0.21, P <.001	rs = 0.13, P= 0.08	rs = -0.04, P= 0.49	rs = 0.20, P <.001

For imaging biomarkers this was performed without (Full) and with (Partial) adjustment for steatosis.

There was a moderate association between cT1 and histological steatosis (r_s_ = 0.54, P <.001), and a strong association between PDFF and steatosis (r_s_ = 0.68, P <.001); both cT1 and PDFF correlated with the overall NAS (cT1: r_s_ = 0.59, P <.001; PDFF: r_s_ = 0.61, P <.001). There was a moderate correlation between cT1 and PDFF with ballooning grade (cT1: r_s_ = 0.47, P <.001; PDFF: r_s_ = 0.38, P < 0.01), inflammation (cT1: r_s_ = 0.31, P <.001; PDFF: r_s_ = 0.28, P <.001), and for cT1 with fibrosis (cT1: r_s_ = 0.43; P <.0001). There was also a strong association between cT1 and PDFF (r_s_ = 0.66, P <.001).

Given the co-linearity between all variables and the potential for steatosis to dominate the signal, correlations were repeated controlling for steatosis. The resulting partial correlation between both cT1 and PDFF with ballooning indicated that the correlation with cT1 remained (r_s_ = 0.36, P <.001) but the correlation with PDFF was weaker (r_s_ = 0.21, P = 0.03). After correction for steatosis, the correlation with inflammation remained significant for cT1 (r_s_ = 0.17, P <.05) but the correlation with PDFF was no longer significant (r_s_ = 0.13, P = 0.07). Both remained significantly correlated with NAS, although the correlation with PDFF was weaker than for cT1 (cT1: r_s_ = 0.36, P <.001; PDFF: r_s_ = 0.20, P <.001, respectively). Fibrosis remained moderately correlated with cT1 (r_s_ = 0.33; P <.001).

### Difference in cT1 and PDFF Relating to the NAFLD Activity Score and Ballooning

#### cT1

The univariable analysis to predict cT1 adjusting for NAS resulted in a regression model [F_(1,262)_ = 142.2, P <.001] with an adjusted R^2^ suggesting that NAS explained 35% of cT1 variability; the residuals of the model satisfied assumptions of the normal distribution. The coefficient of NAS in the linear regression model suggests that a 2-unit increase in NAS score, has a significant increase in cT1 of 88ms. By way of illustration, an 88ms change in cT1 for a patient moving from NAS 5 to NAS 3 would be equivalent to a drop from 921 to 833 ms.

The above analysis was repeated controlling for liver fat measured using PDFF. The multivariable analysis to predict cT1 from NAS, adjusted for PDFF, resulted in a regression model [F_(1,262)_ = 129.9, P <.001] with an R^2^ suggesting that NAS explained 49% of cT1 variability. Predicted cT1 using the effect estimates of the resultant model indicated an average 44-ms difference in cT1 between two stages of NAS when adjusted for PDFF.

A multivariable analysis was performed with ballooning as the independent variable and revealed a model to predict cT1 [F_(1,262)_ = 73.3, P <.001], with an R^2^ suggesting that ballooning explained 22% of cT1 variability. The coefficient of ballooning in the linear regression model suggested that a 1 unit increase in ballooning has a significant increase in cT1 of 81 ms. This remained significant, but the estimated coefficient was reduced to 44 ms if the model was adjusted for PDFF.

#### PDFF

The univariable analysis to predict PDFF from NAS resulted in a regression model [F_(1,262)_ = 157, P <.001]; however, the residuals of the model did not satisfy assumptions of the normal distribution and thus PDFF data were log-transformed and the model repeated. This resulted in a model [F_(1,261)_ = 123, P <.001] with an R^2^ suggesting that NAS explained 32% of PDFF variability. The coefficient of NAS in the linear regression model suggests that a 2-unit increase in NAS score has a significant relative increase in PDFF of 21.1%. By way of illustration, a 21.1% relative change in PDFF for a patient moving from NAS 5 to NAS 3 would be equivalent to a drop from 15.6% to 12.4%.

A univariable analysis was performed with ballooning as the independent variable and revealed a model to predict PDFF [F_(1,261)_ = 39, P <.001] with an R^2^ suggesting that ballooning explained 13% of PDFF variability. The coefficient of ballooning in the linear regression model suggests that a 1 unit increase in ballooning score, has a significant relative increase in PDFF of 16%.

## Discussion

Our results show positive correlations between the quantitative image–derived biomarkers (QIBs) of cT1 and PDFF, with all histopathological hallmarks of NASH, and between these histological features themselves. This serves to demonstrate the complex interactions between the nature and timing of the pathology in NAFL and NASH, and to demonstrate how increasing levels of hepatic steatosis are associated with more profound hepatocyte injury, that can ultimately result in fibrosis (37), the downstream consequence of NASH linked to poor clinical outcomes (38). Steatosis is the dominant feature for both NAFL and NASH and has the potential to dominate or confound imaging derived metrics. While a change in PDFF is a common endpoint in proof-of-concept Phase 2 NASH studies (31, 32), our study revealed an interesting observation when controlling for steatosis in the correlation analyses. In these analyses, the relationships between the QIBs and histology metrics were all weakened highlighting the contribution of fat to the signal for both cT1 and PDFF. Despite this cT1 still correlated significantly with inflammation, ballooning, and fibrosis; PDFF however was only weakly associated with ballooning and was not linearly related to either inflammation or fibrosis. In order to explore how a difference in disease severity (defined by NAS) relates to a difference in QIBs, we performed linear regression modeling. NAS was able to significantly predict cT1 and PDFF, with parameter estimates for a difference between two points in the NAS equivalent to 88 ms in cT1 and a 21% relative difference in PDFF. These results are in reasonably close agreement with observations from two previous longitudinal NASH clinical trials (30, 33). Given the resolution of NASH is another commonly used endpoint in clinical trials, which is defined as the absence of fatty liver disease and a score of 0–1 for inflammation and 0 for ballooning, we set out to explore the estimate change in QIB related to a one-point change in ballooning. The results suggested an 81 ms difference in cT1 or a 16% relative difference in PDFF was equivalent to the difference between one point in ballooning.

While PDFF is not strongly associated with disease activity and has an inverted-u shaped relationship with fibrosis, the positive associations between the histopathological measures in non-cirrhotic NASH translate into patients with more steatosis being more likely to exhibit characteristics of advanced features of NASH (39); and thus, changes in hepatic steatosis may be correlated with changes in other histological endpoints. This phenomenon has recently been observed in a trial of resmetirom, a highly selective thyroid hormone receptor β (THR-β) agonist [(40), NCT03900429] where it was observed that fat reduction, as measured by week 12 MRI-PDFF, predicted NASH resolution on biopsy in 64% of cases at week 36. In addition, the authors also reported that higher fat reduction (>50%) was correlated with a greater than 60% likelihood of NASH resolution with fibrosis reduction. It is likely that the sensitivity of PDFF to change in NASH status is driven more by the indirect and complex interplay between the mechanisms that result in a downstream change in disease activity when liver fat is reduced, rather than the change in activity itself. Regardless, the above results demonstrate the potential utility for both QIBs to detect a meaningful change in NAS and ballooning and confirm the observation regarding the relative difference in PDFF of around 30% for a two-point change in NAS. Furthermore, it demonstrates the added value of the cT1 measurement as a biomarker of disease activity and fibrosis. This relationship is highlighted when steatosis is controlled for in the partial correlations.

Any interpretation of what constitutes a meaningful change in a biomarker must of course also consider the intended use population and the technical performance, in particular the variability expected across repeated measures with no change in underlying pathology. Both QIBs have been used in variety of NAFLD and NASH studies (e.g., Regenerate, NCT02548351; NGM282, NCT02443116, Maestro-Nash, NCT03900429) and subjected to rigorous test-retest performance testing (17, 18), the performance of particular metrics reported in this analysis were published previously (17). The repeatability coefficients for test-retest of the Liver*MultiScan* reported QIBs, which represent the variation that may be expected across repeated measures were 46 ms for cT1 and 0.8% (absolute) for PDFF. This is lower variation than has been reported previously for PDFF (18) likely due to standardization of acquisition methods and advances in post-processing employed in Liver*MultiScan* software. Techniques that have continued to develop since the data acquisition used in this analysis, are the implementation of Liver*MultiScan* ^®^ IDEAL (Iterative Decomposition of water and fat with Echo Asymmetry and Least-squares estimation) (41) and magnitude only reconstruction (MAGO) (42) post-processing techniques. By comparison, while the financial and human costs of liver biopsy in clinical trials are high, the biggest problem is the lack of precision. There is considerable discordance between even expert pathology readers in NASH clinical trials, with expert inter-rater agreement for steatohepatitis diagnosis reported as 0.66 and 0.52 for the NAS (43). While there is an abundance of data emerging for the utility and interpretation of both QIBs in NAFLD and NASH, it should be acknowledged that as part of the metabolic syndrome, other factors such as gender may be contributing to the signal. The overall effect of age and gender on cT1 values has been evaluated previously using data from the UK biobank imaging study with results revealing these effects are minimal. This study reported a trend for cT1 to be lower in women, although not significantly, and also lower in older compared to younger women, again not significant (44).

In terms of the different utility of the QIBs for detecting clinically meaningful change with pharmacological interventions, both the correlation analysis and the literature suggests PDFF (15) and cT1 (21) are both sensitive to modulation of liver fat. However, steatosis is also closely associated with the other histopathological hallmarks of the disease and it is very difficult to dissociate them. cT1 may offer an advantage over PDFF as an endpoint in NASH clinical trials due to the fact that it is also independently associated with inflammation and fibrosis. These are often the features of greatest interest to the physician and healthcare communities because they correlate the most to clinical outcomes, thus are driving research into emerging pharmacotherapies regarding these specific mechanisms of action [e.g., Farnesoid X receptor (FXR) agonists, FG19, and FG21 analogs, THRb and PPARδ agonists]. Thus, combining the information from both PDFF and cT1 is likely to be superior to either on their own for understanding the treatment response dynamics, particularly when interested in more than the movement of fat from the liver.

Limitations of this study were that the estimates for the difference in biomarker measurement related to histological changes were derived from cross-sectional rather than longitudinal data and that the histological data were obtained independently, without central reads. Given the known large inter-rater variability for granular histologic data such as inflammation and ballooning, there is a possibility for discordance between readers. This however was mitigated to some extent as each study had at least dual read with consensus review. Also, while the combined sample size is substantial, it does not cover all possible combinations of the NAS and fibrosis scores meaning the linear relationships may not be accurately reflected. Despite these limitations, it is encouraging that these data closely reflect the observations from prospective interventional clinical trials using PDFF (32, 45) and cT1 (30, 31, 46). The availability of longitudinal data from ongoing clinical trials will be extremely valuable in validating the conclusions drawn in this study and will hopefully be made available by large research consortia in the USA and Europe [e.g., the Non-Invasive Biomarkers of Metabolic Liver Disease (NIMBLE) project and the Liver Investigation: Testing Marker Utility in Steatohepatitis (LITMUS) project]. Furthermore, the inherent difficulties with the histopathological interpretation of liver tissue may also be addressed in the future by emerging digital pathology techniques.

## Conclusions

In summary, both cT1 and PDFF show correlations with the histopathological features of NASH and show potential as non-invasive endpoints in NASH trials to detect a relevant pharmacodynamic response. A cT1 difference of 88 or 81 ms is related to a two-point change in NAS and a one-point decrease in ballooning, respectively. Similarly, a relative difference of 21% in PDFF is related to a two-point change in NAS, and a relative difference of >16% to a one-point change in ballooning. As PDFF is largely dominated by steatosis, cT1 shows superiority when the focus is on changes in inflammation and/or fibrosis, and thus using both in combination may provide more granularity for distinguishing specific treatment effects.

## Data Availability Statement

The data analyzed in this study is subject to the following licenses/restrictions: The data is owned by the sponsors for each study. Requests to access these datasets should be directed to andrea.dennis@perspectum.com.

## Ethics Statement

The studies involving human participants were reviewed and approved. All studies were conducted in accordance with the ethical principles of the Declaration of Helsinki 2013 and Good Clinical Practice Guidelines. The RIAL study was approved by the institutional review departments at the University of Oxford and by the National Review Ethics Service (South Central; Ref: 11/H0504/2). The CALM study was approved by the institutional review departments at the University of Birmingham and by the National Review Ethics Service (West Midlands – The Black Country; Ref: 14/WM/0010). The prevalence study was approved by the institutional review at the Brooke Army Medical Centre. The RIAL study was registered with clinicaltrials.gov (NCT01543646) and was sponsored by the University of Oxford. The CALM study was registered with the International Standard Randomized Controlled Trial Number registry (ISRCTN39463479) and the National Institute of Health Research portfolio (15912). The study sponsor was the University of Birmingham. The Prevalence study was registered with clinicaltrials.gov (NCT03142867) and was sponsored by The Geneva Foundation. All participants provided their written informed consent to participate in these studies.

## Author Contributions

AD performed the analysis and wrote the manuscript. MK supported analysis and contributed to the manuscript. CF developed the LMS processing algorithm and supported data analysis. SM contributed to statistical analysis. JF, GH, MP, and SH were all investigators in the trials from which the data were pooled, were involved in study design and data analysis and data interpretation, and reviewed and edited the manuscript. MC, RB, and AS all reviewed and contributed to the manuscript. All authors contributed to the article and approved the submitted version.

## Funding

The RIAL and NICOLA studies were supported by grants from the Oxford NIHR Biomedical Research Centre and the Oxford Health Services Research Committee (OHSRC). The CALM study was academic-led, sponsored by the University of Birmingham and funded by Innovate UK (Enhancing In Vivo Imaging for Stratified Medicine) Award (TSB Ref: 31620-234143). The Prevalence study was sponsored by The Geneva Foundation and funded by EchoSens.

## Conflict of Interest

Perspectum Ltd is a privately funded commercial enterprise that develops medical devices to address unmet clinical needs, including LiverMultiScan^®^. RB is the CEO and founder of Perspectum. AD, SM, CF, and MK are employees of Perspectum. MP is a shareholder in Perspectum.

The remaining authors declare that the research was conducted in the absence of any commercial or financial relationships that could be construed as a potential conflict of interest.

## Abbreviations

MRI, magnetic resonance imaging; NAS, NAFLD activity score; PDFF, proton density fat fraction; NAFLD, non-alcoholic fatty liver disease; NASH, non-alcoholic steatohepatitis; shMOLLI, shortened modified look-locker inversion recovery; QIB, quantitative imaging biomarker; ELF, Enhanced Liver Fibrosis test; PRO-C3, pro-peptide of type III collagen; IDEAL, Iterative Decomposition of water and fat with Echo Asymmetry and Least-squares estimation; MAGO, Magnitude only processing; FXR, Farnesoid X receptor; FG19 and FG21 analogs, Fibroblast growth factor; THRb, thyroid hormone receptors; PPARδ agonists, Peroxisome proliferator-activated receptors.
